# How We Manage Patients with Indolent B-Cell Malignancies on Bruton’s Tyrosine Kinase Inhibitors: Practical Considerations for Nurses and Pharmacists

**DOI:** 10.3390/curroncol30040322

**Published:** 2023-04-18

**Authors:** Shannon Nixon, Dominic Duquette, Sarah Doucette, Jean-Francois Larouche

**Affiliations:** 1 Malignant Hematology, Princess Margaret Cancer Centre, Toronto, ON M5G 2M9, Canada; 2 Department of Pharmacy, Hôpital de l’Enfant-Jésus, CHU de Québec-Université Laval, Quebec City, QC G1J 1Z4, Canada; 3 Impact Medicom Inc., Toronto, ON M6S 3K2, Canada; 4 Department of Medicine, CHU de Québec-Université Laval, Quebec City, QC G1J 1Z4, Canada

**Keywords:** Bruton’s tyrosine kinase inhibitor, non-Hodgkin lymphoma, chronic lymphocytic leukemia, safety, adverse event, drug interactions, ibrutinib, acalabrutinib, zanubrutinib

## Abstract

The most common forms of B-cell malignancy, non-Hodgkin lymphoma (NHL) and chronic lymphocytic leukemia (CLL), have seen a drastic shift in the treatment landscape over the last two decades with the introduction of targeted agents. Among them are Bruton’s tyrosine kinase (BTK) inhibitors, which have demonstrated excellent efficacy in indolent B-cell NHLs and CLL. Although BTK inhibitors are generally thought to be more tolerable than chemoimmunotherapy, they are associated with a unique safety profile including varying rates of rash, diarrhea, musculoskeletal events, cardiovascular events, and bleeding. Ibrutinib was the first BTK inhibitor to gain a Health Canada indication, followed by second-generation BTK inhibitors acalabrutinib and zanubrutinib, which have better safety profiles compared to ibrutinib, likely due to their improved selectivity for BTK. As BTK inhibitors are oral agents given continuously until disease progression, long-term adverse event (AE) monitoring and management as well as polypharmacy considerations are important for maintaining patient quality of life. This paper intends to serve as a reference for Canadian nurses and pharmacists on dosing, co-administration, and AE management strategies when caring for patients with indolent B-cell NHL or CLL being treated with BTK inhibitors.

## 1. Introduction

B-cell malignancies are a diverse group of lymphoproliferative neoplasms originating from B-cells at different stages of differentiation. Non-Hodgkin lymphoma (NHL) and chronic lymphocytic leukemia (CLL) are the most common forms of B-cell malignancy, with age standardized incidence rates of 25 and six cases per 100,000 people per year in Canada, respectively [1]. There are over 40 subtypes of NHL defined by distinct histological and immunophenotypic characteristics, the majority of which originate from B-cells (~85% in North America) and a minority originating from T-cells and natural killer (NK) cells [2,3]. While CLL is a single entity, it is biologically similar to small lymphocytic lymphoma (SLL), a subtype of NHL, and is often considered under the B-cell NHL umbrella [2].

Many B-cell NHLs have an indolent disease course such as marginal zone lymphoma (MZL), CLL, and follicular lymphoma (FL), which have 5-year relative survival rates of 84%, 88%, and 90%, respectively [4,5,6]. Indolent NHLs are not curable, however, they are associated with a long survival due to recent advances in therapy. Active surveillance is reasonable in the majority of asymptomatic patients, while treatment is indicated in early or advanced stages for those who develop symptoms, and is often repeated over the course of their disease [7].

The treatment landscape for indolent B-cell NHLs has changed drastically over the last two decades due to an increased understanding of disease biology, which has led to the development of effective targeted therapeutics. This includes inhibitors of Bruton’s tyrosine kinase (BTK), a member of the TEC family of protein kinases that plays a role in signal transduction downstream of the B-cell receptor [8]. With its critical role in normal B-cell function and reported increased expression and activation in several B-cell NHLs, BTK has become an attractive drug target for these neoplasms [8].

In 2014, ibrutinib was the first BTK inhibitor to gain Health Canada approval based on the RESONATE trial, which demonstrated superior progression-free survival (PFS) and overall survival (OS) compared to ofatumumab (an anti-CD20 monoclonal antibody) in patients with relapsed CLL [9]. Notably, the benefit of ibrutinib also applied to patients who had deletions in chromosome 17 (del[17p]), a subgroup who historically respond poorly to chemoimmunotherapy. Since then, ibrutinib has received several other indications in CLL as well as for mantle cell lymphoma (MCL), MZL, and Waldenstrom’s macroglobulinemia (WM). Second-generation BTK inhibitors, acalabrutinib and zanubrutinib, have also received Health Canada indications in some of these NHL subtypes, with first indications approved in 2019 and 2021, respectively [10,11,12] (Table 1).

Bruton’s tyrosine kinase inhibitors may be used in the first-line (CLL and WM) or relapsed setting (CLL and iNHL), depending on access within jurisdictions. Phase 3 trials in CLL have demonstrated improved progression-free survival with BTK inhibitors over standards of care at the time of study initiation [16,17,18,19,20]; however, they have not yet been directly compared to fixed-duration venetoclax-based regimens that have also demonstrated improved efficacy over standards of care in CLL [21,22]. As BTK inhibitors are given orally, they may be a suitable option for patients who have difficulty travelling to cancer centers for treatment or who may wish to reduce public exposure to prevent infections. They also have a favorable toxicity profile compared to chemoimmunotherapy, with lower rates of hematological toxicity and infection, making them an attractive option for patients who are older or have multiple comorbidities. This represents a significant proportion of patients with NHL and CLL, as the median age of diagnosis is 67–70 years [6,23].

Although BTK inhibitors are generally better tolerated than chemotherapy-based regimens, these oral agents are associated with a unique adverse event (AE) profile that includes varying rates of rash, diarrhea, arthralgias, myalgias, bleeding, and cardiovascular events. As BTK inhibitors are given continuously until disease progression, appropriate prevention and management of these AEs is particularly important to maintain patient quality of life and to allow patients to continue therapy. Long-term AE monitoring and polypharmacy considerations requiring the involvement of multiple members of the health care team are also needed. This paper discusses the dosing, co-administration considerations, and AE management strategies for BTK inhibitors from a Canadian perspective. It intends to serve as a reference for nurses and pharmacists involved in the care of patients with indolent B-cell NHL or CLL who are being treated with BTK inhibitors.

## 2. Pharmacokinetics and Dosing

Ibrutinib, acalabrutinib, and zanubrutinib are all orally available BTK inhibitors that function by forming irreversible covalent bonds with the C481 residue in the ATP binding domain of BTK, thus inactivating its kinase function. The dosing for each BTK inhibitor was chosen based on the optimal BTK occupancy (>95%) as phase I dose escalation studies did not find any dose-limiting toxicities [24,25,26] (Table 2). While ibrutinib indications recommend a once-daily dose of 420 mg (CLL and WM) or 560 mg (MCL or MZL) until disease progression, acalabrutinib is given at a recommended dose of 100 mg twice-daily given this dosing scheme produced superior BTK occupancy compared to 100–400 mg once-daily. Zanubrutinib 160 mg twice-daily was established as the recommended phase 2 dose based on more sustained BTK occupancy in the lymph nodes at this dose. However, zanubrutinib is approved at a dose of either 320 mg once-daily or 160 mg twice-daily, since both doses can achieve BTK occupancy greater than 95% with no difference in efficacy or safety between the once- and twice-daily dosing cohorts observed in patients with CLL [26].

**Figure 1:**
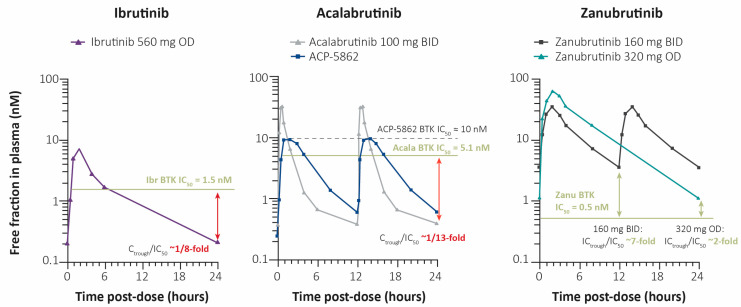
Mean plasma concentration over time for ibrutinib, acalabrutinib, ACP-5862 (major metabolite of acalabrutinib), and zanubrutinib. Adapted with permission from Clinical pharmacology and PK/PD translation of the second-generation Bruton’s tyrosine kinase inhibitor, zanubrutinib by Constantine S. Tam, Ying C. Ou, Judith Trotman & Stephen Opat © [27] The Author(s) 2021 taken from Expert Review of Clinical Pharmacology © 2021, 14:11, 1329–1344 © Taylor & Francis Ltd 2021, reprinted by permission of the publisher, Taylor & Francis. Acala, acalabrutinib; BID, twice-daily; BTK, Bruton’s Tyrosine Kinase; C, concentration; Ibr, ibrutinib; IC50, half maximal inhibitory concentration; OD, once-daily; Zanu, zanubrutinib. BTK Selectivity.

Based on pharmacokinetic studies, the mean area under the concentration–time curves over 24 h for ibrutinib 420 mg once-daily, acalabrutinib 100 mg twice-daily, and zanubrutinib 160 mg are approximately 732 ng·h/mL (71% coefficient of variation [CV]), 1843 ng·h/mL (38% CV), and 2099 ng·h/mL (42% CV), respectively [10,11,12] (Table 2). A dose-proportional increase in exposure for all three BTK inhibitors was observed, with no drug accumulation. Additional pharmacokinetic parameters for each BTK inhibitor are presented in Table 2 and Figure 1.

**Figure 2:**
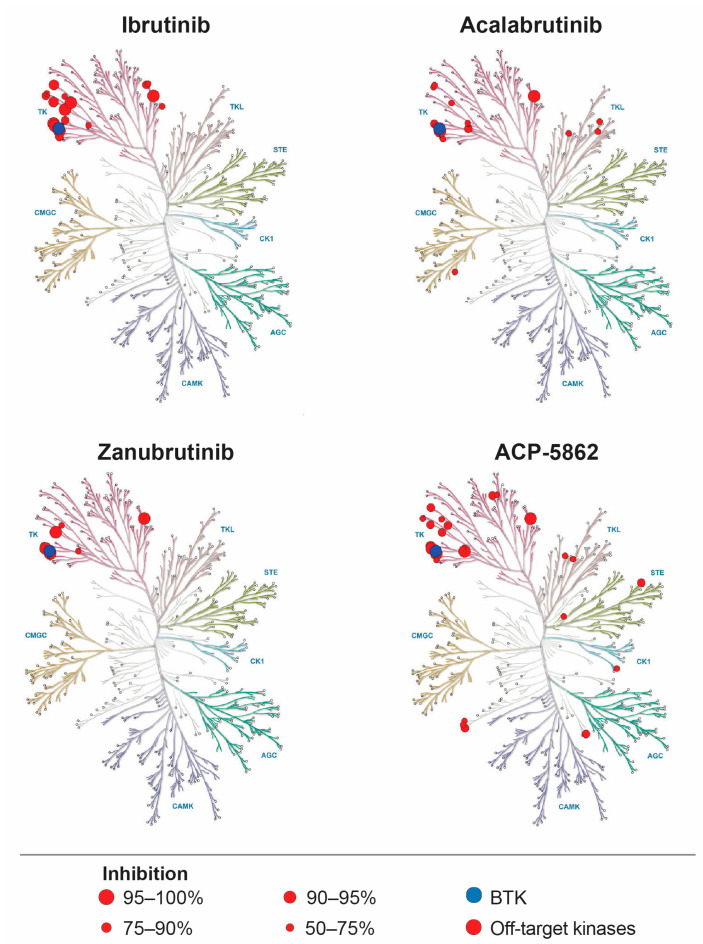
Kinase selectivity of ibrutinib, acalabrutinib, ACP-5862 (major metabolite of acalabrutinib), and zanubrutinib. Adapted with permission from Figure 2 in Shadman et al. (2021) [28]. Kinase selectivity assay was performed by Reaction Biology Corp. at 100x the IC50 value against BTK for ibrutinib (0.32 nM), acalabrutinib (24 nM), ACP-5862 (63 nM), and zanubrutinib (0.71 nM).

The BTK inhibitors differ in their selectivity for BTK and interactions with off-target kinases, which may contribute to differences in the safety profile between the agents. In an assay assessing the percent inhibition of 370 kinases by ibrutinib, acalabrutinib, and zanubrutinib at 100 times their IC50 values, ibrutinib was found to inhibit the most off-target kinases by 50% or more [28] (Figure 2). This study also assessed the inhibitory ability of the major active metabolite for acalabrutinib (ACP-5862), which has a 2–3-fold higher exposure compared to the parent molecule and is 50% less potent at inhibiting BTK [11]. The acalabrutinib metabolite showed greater than 50% inhibition of more off-target kinases than ibrutinib; however, the major metabolite of ibrutinib, which has 2–3 fold higher exposure than the parent molecule and is 15 times less potent, was not assessed [12,28].

Better selectivity of next-generation BTK inhibitors has translated into improved safety outcomes for patients in clinical trials. In head-to-head trials against ibrutinib, both acalabrutinib and zanubrutinib demonstrated a decrease in adverse events of interest, particularly of atrial fibrillation, and a decreased rate of discontinuation [29,30,31]. Based on data showing a worse toxicity profile for ibrutinib and a black box warning for sudden cardiac deaths, international guidelines recommend the use of second generation BTK inhibitors over ibrutinib for patients with CLL [32]. A further rationale for prioritizing zanubrutinib over ibrutinib comes from the updated analysis of the phase 3 ALPINE trial comparing ibrutinib to zanubrutinib in patients with relapsed/refractory CLL, where zanubrutinib demonstrated higher overall response rates (83.5% vs. 74.2%) and a statistically significant improvement in progression-free survival (24-month PFS: 78.4% vs. 65.9%; hazard ratio [HR]: 0.65; 95% confidence interval [CI]: 0.49–0.86; *p* = 0.002) compared to ibrutinib [29]. This was not the case in the phase 3 ELEVATE-RR study in relapsed/refractory CLL, as a similar efficacy for acalabrutinib and ibrutinib were reported with a median PFS of 38.4 months in both arms (HR 1.00; 95% CI: 0.91–1.27) [31].

## 3. Drug Interactions with BTK Inhibitors

Ibrutinib, acalabrutinib, and zanubrutinib are metabolized in the liver, primarily by CYP3A enzymes, which has implications for drug co-administration. It has been suggested that the dose of any BTK inhibitor be reduced if it is being co-administered with moderate CYP3A inhibitors [10,11,12] (Table 3). Although it is suggested to avoid the co-administration of strong CYP3A inhibitors with ibrutinib and acalabrutinib, a reduced dose of zanubrutinib may be given with strong CYP3A inhibitors. Ibrutinib, acalabrutinib, and zanubrutinib should not be given with strong CYP3A inducers as this may reduce BTK inhibitor exposure. In a recent retrospective cohort study of 642 patients with CLL receiving ibrutinib therapy in Ontario, Canada, concomitant CYP3A inducers were identified as a significant risk factor for death on multivariable analysis, with CLL being the most common known cause of death [33]. Variations in CYP3A genotype have been associated with altered metabolism for several non-cancer drugs and could alter BTK inhibitor metabolism [34]. However, testing for the CYP3A genotype is not routinely performed for patients with lymphoma, and clinical studies are needed to understand the potential value of CYP3A genotyping in this setting.

Co-administration of proton pump inhibitors (PPIs) is not recommended with the current capsule formulation of acalabrutinib that is available in Canada as its solubility decreases with increasing pH [11]. This is notable, given that PPIs were prescribed in approximately one third of Canadian seniors in 2016 [36]. The product monograph states that antacids or H2-receptor antagonists may be considered as alternative acid-reducing agents, with acalabrutinib being given 2 h prior to their administration. However, this may not be sufficient to prevent drug interactions as the co-administration of calcium carbonate has been reported to decrease the AUC of acalabrutinib by 53% due to the pH-related reduction in bioavailability [11]. In addition, the acid-reducing activity of H2 antagonists can last for more than 10 h, which can be challenging to coordinate with the twice-daily dosing of acalabrutinib.

All BTK inhibitors are associated with an increased risk in bleeding, therefore caution should be taken when co-administrating with other medicines that may increase bleeding risk. This includes aspirin, anti-inflammatory drugs, anticoagulants, antiplatelets, and certain supplements (e.g., fish oil, flaxseed, vitamin E, omega-3 fatty acids, ginkgo biloba). Many of the clinical trials for BTK inhibitors excluded the use of vitamin K antagonists (e.g., warfarin) due to an association of increased risk of major hemorrhage for patients taking warfarin concurrently with ibrutinib in early clinical trials [37]. The phase 3 SEQUOIA trial, comparing zanubrutinib against bendamustine-rituximab in patients with relapsed CLL, did not have anticoagulant restrictions; however, only four patients receiving concurrent warfarin were enrolled [20,38]. Other antiplatelet agents were allowed in the ASPEN (zanubrutinib vs. ibrutinib in relapsed WM), ALPINE (zanubrutinib vs. ibrutinib in relapsed CLL), and ELEVATE-RR (acalabrutinib vs. ibrutinib in relapsed CLL) trials [29,31,39].

## 4. Considerations for Renal and Hepatic Impairment

The frequency and severity of renal and liver impairment increases with age and must be considered prior to starting any new therapy. BTK inhibitors do not appear to impact kidney function and can be safely given to patients with mild to moderate renal impairment [10,11,12]. The product monograph for ibrutinib recommends that a reduced dose of ibrutinib be given to patients with mild hepatic impairment (Child–Pugh class A) and it should be avoided in patients with moderate and severe hepatic impairment (Child–Pugh classes B and C) [12]. However, for both acalabrutinib and zanubrutinib, patients with mild and moderate hepatic impairment may receive therapy without dose modifications [10,11].

## 5. Adverse Events: Frequency and Management

The effect of adverse events on patients taking BTK inhibitors can range from a minor nuisance to significantly impacting quality of life and daily activities. They can also range from mild in severity to potentially life-threatening. Adverse events that occur frequently at a low grade with all BTK inhibitors include diarrhea, fatigue, rash, musculoskeletal events, and bruising/bleeding. Although these AEs are not usually life-threatening, they can distress patients and have frequently led to treatment discontinuation in clinical trials and real-world studies [40,41,42]. Other AEs associated with BTK inhibitor therapy such as atrial fibrillation, hypertension, major hemorrhage, cytopenia, and infection require immediate intervention and have the potential for life-threatening consequences.

Some of these AEs are thought to result from off-target inhibition of protein kinases and show a trend for decreased incidence with more selective second generation BTK inhibitors compared with ibrutinib. Although some AEs may occur at a lower frequency with second generation BTK inhibitors, they can still occur. Thus, appropriate prevention, monitoring, and management of AEs are needed for patients receiving any BTK inhibitor. The following section describes the frequency, timing, and management strategies for common low-grade AEs and other AEs of interest with BTK inhibitors.

### 5.1. Frequent Low-Grade Adverse Events

#### 5.1.1. Diarrhea

Diarrhea related to BTK inhibitor therapy is generally mild (grade 1 or 2, defined as an increase of 1–3 or 4–6 stools per day over baseline, with some limitations to instrumental activities of daily living) and may be accompanied by abdominal cramping [43]. It has been reported to occur in up to half of patients receiving ibrutinib in clinical trials (Table 4) and has consistently been reported at a lower frequency for acalabrutinib and zanubrutinib in head-to-head trials against ibrutinib [29,30,31]. It is postulated that the decrease in diarrhea with second generation BTK inhibitors may be partly explained by the reduced off-target inhibition of EGFR with these agents compared to ibrutinib, given that diarrhea is a class-effect of EGFR inhibitors [44].

Diarrhea generally occurs within the first 6 months of treatment and resolves within 1–2 weeks without dose modifications [56]. Grade 3 diarrhea (≥7 stools per day over baseline or need for hospitalization) is rare with BTK inhibitor therapy. In these cases, BTK inhibitors may be paused until diarrhea is reduced to grade 2 or lower. The drug can then be re-initiated at the same dose or the dose can be reduced if severe diarrhea recurs [56].

Given the frequency of diarrhea, patients should be educated on management strategies before starting therapy. All events of diarrhea should be assessed by a health care provider to determine the likely etiology, severity, and effect on patient quality of life, which will inform management [57]. Diarrhea assessment should consider evaluation for common pathogens (e.g., *clostridioides difficile*) that can be treated following local guidelines [58]. If abdominal discomfort or pain is present, computed tomography (CT) scan or X-ray should be considered to rule out colitis or overflow diarrhea caused from previous constipation, respectively, as anti-diarrheal agents would not be recommended in these settings [57].

For diarrhea impacting quality of life, pharmacologic intervention may be used as directed by a physician, pharmacist, or nurse practitioner. Loperamide is a frequently used first-line choice for managing diarrhea caused by systemic anti-cancer therapies [57,58]. In refractory cases, diphenoxylate/atropine, opioids, and octreotide may be considered [57]. If diarrhea causes the perianal area to become excoriated, topical barrier or medicated (e.g., corticosteroid) creams can help relieve discomfort.

To avoid dehydration, patients should be advised to increase their intake of non-caffeinated, non-carbonated, and non-alcoholic clear fluids [57,58]. A commercial or homemade oral rehydration solution (1/2 teaspoon salt and 6 teaspoons sugar in 1 L tap water) can also be recommended. Meals can be eaten in smaller portions, more frequently, and should focus on high-calorie foods. Certain foods should be avoided including ones that are high in insoluble fiber (e.g., dark leafy greens), high in sugar, high in fat, acidic, spicy, containing lactose, and/or gas-producing. A BRAT diet (banana, rice, applesauce, toast) may be helpful [58].

#### 5.1.2. Rash and Other Dermatologic Events

In BTK inhibitor trials, rash has been reported in up to 17% of patients (Table 4). Like diarrhea, rash is a common adverse event related to EGFR inhibitors, and its occurrence in patients on BTK inhibitor therapy may be partly explained by the off-target inhibition of EGFR. Rates of rash appear similar between BTK inhibitors in direct comparison trials; however, in clinical trials of ibrutinib-intolerant patients with B-cell malignancies, more than half of the patients who discontinued ibrutinib due to rash did not have a recurrence of this AE on acalabrutinib or zanubrutinib [41,42]. It has also been suggested that rashes occurring in the first few weeks of BTK therapy could be caused by the transient lymphocytosis that occurs in many patients following BTK inhibitor initiation (see hematological toxicities below) [44].

Several types of dermatologic events can occur including early-onset, pruritic rashes, mild petechial rashes (see section on bleeding below), acne-like rashes, eczema-like rashes, and nail changes [59]. Most of these rashes are mild and resolve within a month without dose modifications, although a temporary pause in BTK inhibitor treatment may be useful for grade 3 rashes (covering >30% of the body surface area and impacting self-care activities of daily living) [59,60].

Early-onset palpable, pruritic rashes are common and vary in clinical presentation [59]. In most cases, a dermatology referral is recommended and the rash may be managed with topical corticosteroids and antihistamines [61]. Acne-like rashes (folliculitis) have been reported early in the course of BTK inhibitor therapy and are frequently associated with staphylococcus superinfection [61]. Management should be based on the findings from bacterial and mycological cultures. Eczema-like rashes can also occur, although the rate of occurrence is unclear [61]. Related pruritis can be managed with cold compresses and/or soaking in a lukewarm bath with colloidal oatmeal [62]. Frequent use of alcohol- and fragrance-free moisturizers are also helpful in relieving or preventing skin dryness. These should be applied after washing or showering [62].

Nail changes (including brittleness, separation from nail bed, ridging, depressions, pitting, and splintering) may occur in approximately 60% of patients on progressive BTK inhibitor treatment [63]. This is thought to be caused by BTK inhibitor-mediated disruption of disulfide bonds between cysteine residues, which disrupt the keratin structure [63]. Preventative measures can be taken to avoid or reduce the severity of nail toxicity [61]. These include wearing wide-fitting shoes to remove pressure on nails and trimming nails regularly. Topical solutions (e.g., ointments, hydrosoluble nail lacquer, poly-ureaurethane) and biotin supplementation may be used to manage nail effects [61,63].

Certain symptoms associated with severe rash can signal a severe immune-mediated drug reaction that may require treatment discontinuation. These include fever, facial edema, cutaneous detachment with blisters or mucosal erosions, signs suggestive of Steven–Johnson syndrome, exfoliative rash, pustules, lymphadenopathy, or laboratory abnormalities [61]. Given that patients with B-cell malignancy are immunosuppressed, viral reactivation should also be investigated as a potential cause for widespread rash.

#### 5.1.3. Musculoskeletal Events

Adverse events affecting the musculoskeletal system such as joint pain (arthralgia), muscle pain (myalgia), and muscle spasms are common in patients taking BTK inhibitors. Grade 1/2 arthralgia, muscle spasms, and myalgia are reported in about 20%, 10–15%, and <10% of patients receiving BTK inhibitors, respectively. In head-to-head trials, rates of arthralgia were similar for zanubrutinib compared to ibrutinib and were slightly decreased for acalabrutinib compared to ibrutinib [29,30,31]. In addition, in clinical trials of ibrutinib-intolerant patients, the majority of patients who discontinued ibrutinib due to arthralgia did not have a recurrence of this AE on acalabrutinib or zanubrutinib treatment [41,42]. Muscle spasms have also been reported more frequently with ibrutinib compared to second generation BTK inhibitors in head-to-head trials [29,30,31].

Arthralgia and myalgia typically occur within the first few months of treatment and mild cases often resolve within a few months without dose modifications [56,64,65]. Patients can manage symptoms with pain relievers such as acetaminophen, while anti-inflammatory agents with anti-platelet properties (e.g., ibuprofen) should be avoided as they can further increase the bleeding risk associated with BTK inhibitors. Other complementary management strategies include exercise with a focus on mild stretching and strengthening and the use of hot and cold compresses. The evaluation of electrolyte levels should be performed as supplementation with sodium, potassium, and/or magnesium may improve musculoskeletal pain and spasms in those with deficiencies [66]. For patients with persistent arthralgia (>6 months) that impacts daily activities and quality of life, a dose delay of up to 1 week, followed by a dose reduction can be considered, although this management strategy is anecdotal and efficacy is variable [56].

#### 5.1.4. Headache

Low-grade headaches commonly occur in patients on acalabrutinib treatment (Table 4). They occur infrequently in patients treated with ibrutinib and zanubrutinib, thus are often not reported in publications of clinical trials for these BTK inhibitors. Headaches occur soon after treatment initiation and resolve over a few months. Dose modification is not typically needed as patients can be effectively managed with acetaminophen and/or caffeine [67]. Headache in the setting of thrombocytopenia or anti-coagulation, falls and/or trauma should be investigated to rule-out alternate etiology (e.g., CNS bleeding) [67].

#### 5.1.5. Fatigue

Fatigue is among the most common symptoms reported at diagnosis in many cancer types and is also a common treatment side-effect [43,68]. It may present as tiredness, which is disproportionate to recent activity and impairs activities of daily living or quality of life [68]. It may also be accompanied by decreased concentration/attention; feelings of distress towards being fatigued (e.g., sad, frustrated, irritable), sleep disturbance or sleep being perceived as non-restorative, or decreased motivation or interest in usual activities.

Similar rates of fatigue of any grade among BTK inhibitors were reported in head-to-head trials [29,30,31]. However, the rate of grade 3 fatigue was significantly higher in the acalabrutinib arm compared to the ibrutinib arm in the ELEVATE-RR study (3.4% vs. 0%) [31]. In addition, in clinical trials of ibrutinib-intolerant patients, the majority of patients who discontinued ibrutinib due to fatigue did not have a recurrence on zanubrutinib (8 of 15 patients), while three out of four patients had a recurrence on acalabrutinib [41,42].

Fatigue occurs most frequently in the first year of treatment with BTK inhibitors and is usually self-limited. As early fatigue could be a symptom of underlying disease rather than being drug-related, initial dose modifications are not recommended [69]. If fatigue begins later in treatment or persists, other potential sources of fatigue should be investigated, and drug modification should be considered if fatigue is deemed to be related to BTK inhibitor treatment [69].

Fatigue can be challenging to manage as its etiology is not well-understood. There is currently insufficient evidence to support the benefit of pharmacological intervention in fatigue management [68]. Non-pharmacological interventions that may improve fatigue include energy prioritization, optimizing sleep quality, moderate intensity physical activity (e.g., walking, yoga), cognitive behavioral therapy, attention restoring therapy (e.g., reading, games, music, etc.), and stress reduction strategies.

### 5.2. Other Adverse Events of Interest

#### 5.2.1. Bleeding

Minor bleeding events such as mild petechial rash, bruising, and skin ecchymoses occur in up to 40% of patients on BTK inhibitor therapy. They commonly occur within the first year but can occur at any time over the course of therapy [60]. These dermatologic events are likely related to platelet dysfunction caused by the on-target inhibition of BTK and off-target inhibition of TEC and Src kinases, and is not necessarily associated with platelet count [44,70]. They may resolve spontaneously without intervention but can be managed with ice and topical moisturizers [61,71,72]. Patients should be aware of these minor bleeding events and can be reassured that they have not been associated with increased risk of major hemorrhage [61,73].

Major hemorrhage is a less common but severe complication associated with BTK inhibitor therapy. The mechanism underlying increased bleeding tendency remains unclear, however, major hemorrhage occurs in about 5% of patients on BTK inhibitor therapy at a similar rate between each BTK inhibitor [29,31]. It occurs most often in the first year of therapy, but it can occur at any time over the course of treatment. It can begin anywhere in the body and cause a range of ambiguous symptoms [74]. Patient education on signs and symptoms of hemorrhage play an important role in managing this AE. Patients should be educated on the increased risk of major hemorrhage and to visit the emergency department if they experience signs and symptoms of bleeding including localized pain (e.g., head, chest, abdomen, back), severe bruising, blood in vomit, urine, or stool, shortness of breath, dizziness, altered mental state, thirst, decreased urination, cold, clammy skin, increased heart rate, and decreased blood pressure [74,75].

Risk reduction strategies play an important role in bleeding prevention. Patients should be advised that both prescription and non-prescription drugs and/or supplements can increase bleeding risk, and thus a pharmacist should be informed before the initiation of any new drug or supplement (see section on drug interactions) [76]. Patients should also be informed of the need to hold their BTK inhibitor treatment before and after planned surgery to reduce the risk of bleeding. Product monographs suggest that BTK inhibitors should be stopped 3–7 days before and after a procedure depending on the type of surgery and risk of bleeding [10,11,12]. This is supported by an in vitro analysis demonstrating the full reversal of the anti-platelet effects of ibrutinib after 1 week off therapy [70,77,78].

#### 5.2.2. Hypertension

About 20% of patients on BTK inhibitor therapy will experience new or worsening hypertension. In head-to-head trials, a decreased frequency of hypertension was reported for acalabrutinib compared to ibrutinib, while zanubrutinib showed a similar rate of hypertension to ibrutinib in one study (ALPINE), and a decreased rate of hypertension in another study (ASPEN) [29,30,31]. In addition, in a clinical trial of ibrutinib-intolerant patients, the majority of patients who discontinued ibrutinib due to hypertension did not have a recurrence of this AE on zanubrutinib treatment [42].

Unlike most AEs, which have the highest onset in the first year of BTK inhibitor therapy, the incidence of new onset hypertension increases over the course of treatment [79]. In addition, higher rates of hypertension have been reported in real-world studies, with up to 78% of patients receiving ibrutinib and 49% receiving acalabrutinib having new or worsened hypertension [80,81,82]. Risk factors for the development of new hypertension based on multivariable analysis include prior arrhythmias and Black ancestry [82]. In real-world studies, new or worsened hypertension was also associated with increased risk of major adverse cardiovascular events (MACE) [82]. Although no single class of antihypertensive agent has been associated with the prevention or control of BTK inhibitor-related hypertension, the initiation of any antihypertensive agent was associated with a lower risk of MACE [82].

Given its high frequency, late onset, and association with MACE, hypertension requires vigilant monitoring throughout the course of BTK inhibitor treatment. Patients should be encouraged to check their blood pressure at regular intervals at home, if they have access to a blood pressure monitor, and inform their doctor if their systolic blood pressure is ≥130 mmHg and/or diastolic blood pressure is ≥80 mmHg. This may be an indication for closer monitoring and/or initiation of anti-hypertensive treatment depending on the level of elevation or whether additional cardiovascular risks are present [83,84]. If systolic blood pressure is ≥180 mmHg and/or diastolic blood pressure is ≥120 mmHg, this is considered as a hypertensive crisis, requiring immediate medical care [85]. Elevated blood pressure can be effectively managed with antihypertensive agents without the need for BTK inhibitor dose modifications [56].

#### 5.2.3. Atrial Fibrillation

Atrial fibrillation is among the most common AEs leading to treatment discontinuation on BTK inhibitor therapy. Atrial fibrillation has consistently been reported more frequently in the BTK inhibitor arms than control arms in clinical trials [20,51,52,86,87,88]. In direct comparison trials, atrial fibrillation was reported less frequently with second generation BTK inhibitors than with ibrutinib [29,30,31] (Table 4). Real-world studies suggest that the true rate of atrial fibrillation may exceed that reported in clinical trials [83]. In addition, in a clinical trial of ibrutinib-intolerant patients, the majority of patients who discontinued ibrutinib due to atrial fibrillation did not have a recurrence on zanubrutinib treatment [42].

The rate of new atrial fibrillation events generally remains stable over the course of BTK inhibitor therapy [60]. Several studies have identified potential risk factors for the development of atrial fibrillation during BTK inhibitor therapy including existing cardiac comorbidities, left atrial volume, older age, and being male [89,90]. Patients should be asked about symptoms of arrhythmias including sensations that the heart is racing, fluttering, or pounding in the chest, experiencing unusual shortness of breath, feeling more easily tired, and feeling faint, dizzy, or lightheaded [91]. Health care providers should have a low threshold for cardiac workup in patients reporting symptoms of atrial fibrillation, which should include electrocardiogram, echocardiogram, and the assessment of thyroid stimulating hormone levels [60].

The management of atrial fibrillation can be challenging in patients receiving BTK inhibitors as the treatment of atrial fibrillation often includes anticoagulants, which can exacerbate the bleeding risk already associated with BTK inhibitors. Additionally, rate and rhythm control drugs can interact with BTK inhibitors. Scoring systems including CHA_2_DS_2_-VASc for stroke risk can help determine whether anticoagulation is needed, and the HAS-BLED scoring system can help determine whether the bleeding risk is high and whether alternate cancer therapy should be considered [92]. Consultation with a cardio-oncologist may be needed to determine the optimal treatment course for the patient [93].

If anticoagulation is necessary to reduce stroke risk, newer agents that inhibit factor Xa such as apixaban and rivaroxaban (direct oral anticoagulants, DOACs) are preferred, as even though they are CYP3A4 substrates, they are not expected to have a clinically meaningful increase in the plasma levels when given concurrently with BTK inhibitors [94]. The direct thrombin inhibitor dabigatran should be avoided with ibrutinib as it is a major substrate for P-glycoprotein, which is inhibited by ibrutinib [94]. Rate controlling strategies are typically used in patients with B-cell malignancies, with a preference for beta-blockers as first-line therapy [93], as calcium channel blockers such as diltiazem and verapamil are inhibitors of CYP3A4, which would increase the plasma levels of BTK inhibitors. As a p-glycoprotein substrate, digoxin concentration in plasma may increase if given concurrently with ibrutinib. It is unclear whether BTK inhibitor therapy should be paused until the resolution of atrial fibrillation. The product monograph for ibrutinib recommends a dose hold for grade 3 cardiac arrythmias followed by reinitiation at a reduced dose upon resolution [12]. Treatment should be discontinued if grade 3 arrhythmias recur.

#### 5.2.4. Other Cardiovascular Toxicity

Although atrial fibrillation is the most common MACE reported with BTK inhibitor treatment, ventricular arrhythmias, tachycardia, and sudden deaths have been reported with ibrutinib, acalabrutinib, and zanubrutinib [81,82,95,96,97], although their incidence is rare. Of the BTK inhibitors, ibrutinib has reported the largest number of sudden cardiac deaths on treatment thus far. This led to a letter from Health Canada from the Recalls and Safety Alerts Database on 29 August 2022 and a subsequent update of the product monograph, which now includes fatal and serious cardiac arrhythmias and cardiac failures in the serious warnings and precautions box [12,98]. Case reports and retrospective analyses suggest that a history of atrial fibrillation may be associated with an increased risk of ventricular arrhythmias following ibrutinib treatment [99,100,101].

#### 5.2.5. Hematological Toxicity

Hematological toxicity commonly occurs in CLL both from complications of the disease itself and as a side effect to therapy. Patients can be educated on the importance of routine blood tests to monitor blood cell counts (monthly until blood counts are stable, followed by every 3 months). Cytopenia occurs most frequently within the first year of therapy, and new onset of cytopenia declines at a stable rate thereafter [79]. Head-to-head trials of BTK inhibitors have reported similar rates of anemia, thrombocytopenia, and neutropenia, with the exception of the ASPEN study, which reported a significantly higher rate of grade ≥3 neutropenia in the zanubrutinib versus ibrutinib arm (23.8% vs. 10.2%), although this did not translate into an increased risk of infection [30]. Among the neutropenic patients, granulocyte colony stimulating factor was given more frequently in the zanubrutinib arm than in the ibrutinib arm (47% vs. 31%), and should be considered in patients with grade ≥3 neutropenia [39]. Grade 3/4 neutropenia and thrombocytopenia that is persistent or associated with significant bleeding, fever, or infection requires dose interruptions and rechallenge at the same or a reduced dose after recovery to at least the grade 1 level [10,11,12]. The median time to resolution of neutropenia and thrombocytopenia is approximately 2 weeks, however, a recurrence may happen multiple times, and treatment should be discontinued at the fourth occurrence (Table 5).

Increased absolute lymphocyte count (lymphocytosis) is a well-recognized effect of BTK inhibitor treatment. It commonly occurs within the first few weeks of BTK inhibitor therapy and resolves without intervention at a median time of 3 months [102]. Lymphocytosis is thought to be triggered by BTK inhibitor-mediated disruption of the tumor microenvironment, causing a redistribution of malignant cells from the tissue compartments to the circulating blood [103]. In isolation, it is not associated with disease response or progression nor does it lead to adverse outcomes such as leukostasis [103].

#### 5.2.6. Infection

Patients with CLL on BTK inhibitor therapy are at risk of infections, which may in part be attributed to the biology of the disease itself and may also be partially caused by on- and off-target effects and drug-induced cytopenia [44,69]. Rates of grade ≥3 infections occur at a similar rate among the BTK inhibitors (20–30%), with pneumonia being the most common grade ≥3 infection reported (Table 4) [29,30,31,69]. Infections are most common within the first year of BTK inhibitor therapy and are more common in patients being treated in the relapsed setting [64,69].

Opportunistic infections of particular concern for patients taking BTK inhibitors include fungal infections with aspergillus fumigatus and Pneumocystis jirovecii pneumonia (PJP) given the potential for serious outcomes and the fact that the appropriate treatments for these infections involve strong CYP3A4 inhibitors [60,69]. Patients should be counseled on the signs of infection (e.g., fever over 38 °C, chills, sweating) and instructed to notify their clinic immediately if infection is suspected. Laboratory analysis to identify the source of infection should be performed, with a high suspicion for opportunistic infections and treatment should follow the local resistance patterns. To prevent infection, patients should be educated on proper hand-hygiene and the importance of avoiding close contact with people who are sick. Patients can be encouraged to have all appropriate non-live vaccines after consultation with the treating physician. As there are no clear recommendations for giving infectious prophylaxis with BTK inhibitors, the decision to prescribe prophylaxis should be made based on the individual risk of each patient.

Reactivation of hepatitis B virus (HBV) in patients who have recovered from infection or HBV flare in patients infected with the virus can occur during or after stopping immunosuppressive therapy. Several case reports of severe HBV reactivation on or after BTK inhibitor therapy have been published, whether caused by ibrutinib therapy or prior therapy, and a warning for HBV infection is stated in all BTK inhibitor monographs [10,11,12,104,105,106]. However, there is no formal guidance on how these patients should be managed. Patients should be screened for HBV serology prior to initiating BTK inhibitor treatment and previously infected patients should either be monitored every 3 months for changes in HBV serum markers or given prophylactic antivirals [106].

#### 5.2.7. Hepatotoxicity

Most tyrosine kinase inhibitors including BTK inhibitors can induce hepatotoxicity, possibly through reactive products of drug metabolism [107]. In addition, baseline hepatic impairment may impact metabolism in the liver, increasing the plasma levels of BTK inhibitors, which can induce further damage to the liver. Hepatic impairment can also lead to coagulopathy, which may be additive to the bleeding risk already tied to BTK inhibitor therapy [108]. Severe hepatotoxicity with coagulopathy occurs rarely with BTK inhibitors but has been reported in several case studies of ibrutinib-treated patients, and in one case study in a zanubrutinib-treated patient [107,108]. Liver enzymes should be regularly monitored and if hepatotoxicity is suspected, the BTK inhibitor should be paused while the etiology of the hepatoxicity is assessed.

#### 5.2.8. Second Primary Malignancies

An increased risk of second primary malignancies (SPM) is established for patients with CLL compared to the general population as well as in patients with CLL treated with chemotherapy compared to untreated patients [109]. However, the risk of SPM following BTK inhibitor therapy is not well-characterized. In a single-center retrospective study of 691 patients with CLL who received ibrutinib or acalabrutinib therapy, 9% of patients had developed a SPM (excluding non-melanoma skin cancer) [109]. The overall incidence of SPM was 2.2 times higher for patients treated with BTK inhibitors than what is expected in the general population. This is similar to the standard incidence rate for SPM observed following treatment with fludarabine-cyclophosphamide-rituximab (2.4) and in a large cohort of CLL patients followed prior to the availability of BTK inhibitors (2.2) [110,111]. A significantly increased risk was noted particularly for lung cancer, melanoma, bladder cancer, Merkel cell carcinoma, and salivary gland cancer. Non-melanoma skin cancers were also frequently observed, with a cumulative incidence rate of 15% at 3 years, similar to reports in other CLL cohorts. It remains unclear whether this increase in SPM is treatment-related or linked to a disease-related genetic predisposition. Although there are limitations to this study, it suggests that patients on BTK inhibitors may benefit from cancer screening, particularly for lung cancer and melanoma to support early detection, leading to better prognosis.

Data on SPM with zanubrutinib comes from a pooled safety analysis of 779 patients with B-cell malignancies treated with zanubrutinib in clinical trials [38]. With a median treatment duration of 26 months, SPM was reported in 102 patients (13%) including 82 patients (10.5%) with non-melanoma skin cancer.

## 6. Conclusions

Bruton’s tyrosine kinase inhibitors are effective in many B-cell malignancies including indolent B-cell NHL and CLL, with generally favorable but unique safety profiles. Continuous monitoring and management of AEs (Table 6) as well as potential drug interactions over the course of therapy (Table 3) are critical for maintaining patient quality of life and optimizing patient outcomes. Although BTK inhibitors are now generally given as monotherapy, combination therapies with other anti-cancer agents are being evaluated in clinical trials with great promise as well as next generation reversible BTK inhibitors. As BTK inhibitor therapy evolves, it will be important to understand the differences in the safety profiles between the BTK inhibitor regimens and revisit how patients can best be managed.

## Figures and Tables

**Table 1:** Health Canada indications for Bruton’s tyrosine kinase inhibitors in B-cell malignancies.

Disease	Indications		
	Ibrutinib	Acalabrutinib	Zanubrutinib
CLL	Previously untreated CLL ± anti-CD20 antibody (rituximab or obinutuzumab) Patients with CLL who have received at least one prior therapy ± bendamustine-rituximab	Previously untreated CLL ± Obinutuzumab Patients with CLL who have received at least one prior therapy	Pending ^a^
MCL	Patients with MCL who have received at least one prior therapy	Patients with MCL who have received at least one prior therapy	Patients with MCL who have received at least one prior therapy
MZL	Patients with MZL who have received at least one prior anti-CD20-based therapy	--	Patients with MZL who have received at least one prior anti-CD20-based therapy
WM	Patients with WM ± rituximab	--	Patients with WM

CLL, chronic lymphocytic leukemia; MCL, mantle cell lymphoma; MZL, marginal zone lymphoma; WM, Waldenstrom’s macroglobulinemia. ^a^ Approved as monotherapy for the treatment of CLL by the European Medicines Agency and the U.S. Food and Drug Association and accepted by Health Canada for supplemental new drug submission in CLL [13,14,15].

**Table 2:** Dosing and pharmacokinetics for ibrutinib, acalabrutinib, and zanubrutinib.

Parameter	Ibrutinib	Acalabrutinib	Zanubrutinib
Dose	420 mg OD for CLL/WM 560 mg OD for MCL/MZL ^a^	100 mg BID	320 mg OD or 160 mg BID
Duration	Until disease progression or unacceptable toxicity		
Mean AUC_0–24 h_ (%CV), ng·h/mL	420 mg: 732 (71%) 560 mg: 953 (74%)	Acala: 1843 (38%) ACP-5862 ^b^: 3947 (43%)	160 mg: 2099 (42%) 320 mg: 1917 (59%)
Mean Cmax (%CV), ng·mL	420 mg: 137 (86%) 560 mg: 164 (100%)	Acala: 563 (29%) ACP-5862 ^b^: 451 (52%)	160 mg: 299 (56%) 320 mg: 533 (55%)
Median Tmax	1–2 h	0.9 h	2 h
Mean half-life	4–6 h	1 h	2–4 h

Acala, acalabrutinib; AUC, area under curve; BID, twice-daily; CLL, chronic lymphocytic leukemia; Cmax, maximum concentration; CV, coefficient of variation; MCL, mantle cell lymphoma; MZL, marginal zone lymphoma; OD, once-daily; Waldenstrom’s macroglobulinemia. ^a^ For MCL and MZL the recommended ibrutinib dose is 560 mg once-daily as this dose produced a good response (overall response rate 78%) in the MCL cohort of the initial dose finding studies ^b^ Major active metabolite of acalabrutinib.

**Table 3:** Co-administration considerations for ibrutinib, acalabrutinib, and zanubrutinib.

Co-Administration Considerations	Ibrutinib	Acalabrutinib	Zanubrutinib
Strong CYP3A inhibitors	Avoid ^a^	Avoid	Reduce dose to 80 mg OD
Moderate CYP3A inhibitors	Reduce dose to 280 mg OD	Reduce dose to 100 mg OD	Reduce dose to 80 mg BID
Strong CYP3A inducers	Avoid		
Warfarin/vitamin K antagonists	Avoid	--	--
Grapefruit, Seville oranges, St. John’s wort	Avoid		
Proton pump inhibitors	--	Avoid ^b,c^	--
Renal impairment	No dose adjustment needed for patients with mild/moderate impairment ^d^		
Hepatic impairment	Consider dose reduction to 140 mg for mild impairment Avoid in patients with moderate/severe impairment ^e^	No dose adjustment needed for mild/moderate impairment ^f^ Avoid in patients with severe impairment ^e^	No dose adjustment needed for mild/moderate impairment ^f^ Consider dose reduction to 80 mg BID for severe impairment ^e^
Administer with caution:	Drugs that prolong the PR interval ^g^ Anticoagulants/antiplatelets ^h^ BCRP and P-gp substrates ^i^	BCRP and MATE1 substrates ^j^	--

BCRP, breast cancer resistance protein; BID, twice-daily; CYP3A, cytochrome P450 3A; OD, once-daily; P-gp; P-glycoprotein. ^a^ Reduce ibrutinib dose to 140 mg OD with voriconazole. ^b^ Alternative gastric-acid reducing agents can be considered (e.g., antacid or H2-receptor antagonist 2 h after acalabrutinib); however, this cannot guarantee the prevention of drug interactions. ^c^ A tablet formulation of acalabrutinib that can be administered with any gastric-acid reducing agents including proton pump inhibitors was approved by the U.S. Food and Drug Administration based on data from the ELEVATE-PLUS trial [35]. ^d^ Greater than 30 mL/min creatinine clearance. ^e^ Child–Pugh B or Child–Pugh C ^f^ Child–Pugh A, Child–Pugh B, or total bilirubin between 1.5 and 3 times the upper limit of normal [ULN] regardless of the aspartate aminotransferase [AST] levels. Safety of zanubrutinib has not been evaluated in patients with severe hepatic impairment. ^g^ Ibrutinib has been shown to cause an increase in the PR interval. ^h^ In clinical studies, patients on ibrutinib therapy and concomitant antiplatelet or anticoagulant agents had an increased risk of major bleeding. ^i^ Ibrutinib is an inhibitor of P-gp and BCRP in vitro. It is recommended that narrow therapeutic range BCRP and P-gp substrates be taken at least 6 h before or after ibrutinib. ^j^ Acalabrutinib and its metabolites may increase exposure to co-administered BCRP and MATE1 substrates (e.g., methotrexate and metformin, respectively).

**Table 4:** Frequency and temporal changes in adverse events reported in clinical trials of BTK inhibitors in B-cell malignancies.

Adverse Event	AE Frequency (Range Based on Clinical Trial Data) ^a,c^			Change in AE Prevalence Over Time ^b^
	Ibrutinib	Acalabrutinib	Zanubrutinib	
Diarrhea	24–58%	18–40%	14–16%	Highest prevalence in first year, stable decrease thereafter
Rash	12–17%	6–15%	10–13%	Highest prevalence in first year, gradual decrease thereafter
Headache	20% ^d^	22–51%	11% ^d^	Highest prevalence in first 3 months, drastic decrease thereafter
Arthralgia	16–27%	16–20%	13–15%	Prevalence generally stable over time
Fatigue	13–50%	9–31%	10–19%	Highest prevalence in first year, generally stable thereafter
Atrial fibrillation	9–16%	5–9%	3–8%	Prevalence generally stable or gradually increases over time
Hypertension	16–29%	3–18%	12–22%	Prevalence gradually increases over time
Major hemorrhage	4–12%	1–5%	4–8%	Prevalence generally stable over time
Pneumonia	12–24%	6–19%	5–11%	Prevalence generally stable over time
Grade 3/4 infection	20–45%	15–31%	16–27%	Highest prevalence in first year, gradual decrease thereafter
Grade 3/4 neutropenia	13–25%	11–20%	12–20%	Highest prevalence in first 6 months, stable decrease thereafter
Grade 3/4 thrombocytopenia	3–13%	4–10%	2–6%	Highest prevalence in first 3 months, stable decrease thereafter
Grade 3/4 anemia	3–13%	5–12%	1–5%	Highest prevalence in first 3 months, stable decrease thereafter

^a^ Rates reflect any grade adverse events unless otherwise specified. ^b^ Based on safety analysis of ibrutinib studies [17,45] ^c^ Based on clinical trials evaluating BTK inhibitor monotherapy in CLL and B-NHL where n ≥ 100 and median follow up is ≥24 months [29,30,31,46,47,48,49,50,51,52,53,54,55]. Incidence of adverse events may be higher in the real-world setting. ^d^ Headache not frequently reported for ibrutinib and zanubrutinib, data based on reports from a single study.

**Table 5:** Recommended BTK inhibitor dose modifications for grade 4 hematological toxicity, grade 3 thrombocytopenia with significant bleeding, or grade 3 febrile neutropenia.

Adverse Reaction Occurrence	Dose Modification		
	Ibrutinib	Acalabrutinib	Zanubrutinib
None (starting dose)	420 mg OD or 560 mg OD	100 mg BID	160 mg BID or 320 mg OD
First	Pause until toxicity resolves to ≤grade 1 ^a^, resume starting dose		
Second	Pause until toxicity resolves to ≤grade 1 ^a^, resume at 280 mg or 420 mg OD	Pause until toxicity resolves to ≤grade 1 ^a^, resume starting dose	Pause until toxicity resolves to ≤grade 1 ^a^, resume at 80 mg BID or 160 mg OD
Third	Pause until toxicity resolves to ≤grade 1 ^a^, resume at 140 mg or 280 mg OD	Pause until toxicity resolves to ≤grade 1 ^a^, resume at 100 mg OD	Pause until toxicity resolves to ≤grade 1 ^a^, resume at 80 mg OD
Fourth	Discontinue		

^a^ or resolve to baseline, BID, twice-daily; OD, once-daily.

**Table 6:** Summary of the management and monitoring strategies for BTK inhibitor-associated adverse events.

Co-Administration Considerations	Manage/Prevent	Monitor/Educate
Diarrhea	Dose modifications not required for grade 1/2 events Suggest: - Increase fluid intake - Small frequent meals - Eat low-fiber, high calorie food (BRAT diet) - Topical barrier cream for perianal area - Take anti-diarrheal medication	Evaluate stool for common pathogens If abdominal discomfort is present, consider CT or X-ray to rule out colitis or overflow diarrhea
Fatigue	Dose modification typically not needed Suggest: - Energy prioritization - Improving sleep quality - Physical activity (e.g., walking, yoga) - Limit stress - Cognitive behavioral therapy - Attention restoring therapy (e.g., games, music)	When fatigue occurs later in treatment, patient should be evaluated for other potential causes
Headache	Dose modifications not needed Suggest taking acetaminophen and caffeine	Unresolved and severe headaches should be evaluated at emergency department
Musculoskeletal events	Dose modifications typically not needed for mild cases; may be required for severe and persistent cases Suggest: - Mild stretching/strengthening routines - Hot/cold compresses - Acetaminophen	Evaluate for electrolyte deficiencies and supplement with sodium, potassium, magnesium as needed
Rash	Dose modifications typically not needed for mild cases; may be required for severe and persistent cases Generally requires dermatology referral and treatment with topical corticosteroids and antihistamines Suggestions for pruritis: - Cold compresses - Lukewarm bath with colloidal oatmeal	Monitor for signs of severe rash: - Fever - Facial swelling - Cutaneous detachment with blisters or mucosal erosions - Signs of Steven–Johnson syndrome - Exfoliative rash - Pustules - Lymphadenopathy - Lab abnormalities
Nail effects	Preventative measures: - Avoid repeated trauma and pressure on nails - Trim nails regularly Suggest: - Biotin supplementation - Topical ointments - Hydrosoluble nail lacquer - Poly-urethaneurea	
Hypertension	Effectively managed with antihypertensive agents	Regularly monitor blood pressure at clinic and at home where possible Educate patient to seek emergency care if systolic/diastolic pressure is ≥180 mmHg/≥120 mmHg
Atrial fibrillation	Assess individual stroke/bleeding risk and consult with specialists as needed BTK inhibitor dose should be interrupted for grade ≥3 events	Inquire and educate patients on signs of arrhythmias: - Heart racing, fluttering, pounding - Shortness of breath - Easily tired - Faint, dizzy Teach patients how to take pulse at home
Minor Bleeding (bruising/petechiae)	Can resolve spontaneously without intervention Suggest applying ice and moisturizers to affected areas	Reassure patients that mild bleeding is not a predictor of major hemorrhage
Major Bleeding	Preventative measures: - Assess concomitant medication and reduce non-essential drugs that may contribute to bleeding risk - Pause BTK inhibitor 3–7 days before and after surgical procedure depending on bleeding risk	Educate on signs of hemorrhage: - Localized pain - Severe bruising - Blood in vomit, urine, or stool - Shortness of breath - Dizziness, altered mental state - Thirst, decreased urination - Cold, clammy skin - Increased heart rate - Decreased blood pressure
Hematological toxicity	Consider supportive care with G-CSF for patients with grade ≥3 neutropenia For grade 3/4 neutropenia and thrombocytopenia that is persistent, or is associated with significant bleeding, fever, or infection, follow dose modifications outlined in the product monographs	Monitor blood counts monthly until blood is stable and every 3 months thereafter
Infection	Preventative measures: - Give appropriate non-live vaccines and prophylactic therapies prior to therapy initiation, in consultation with physician Management - Drink plenty of fluids - Adhere to prescribed medications - Contact clinic if symptoms worsen/do not improve	Educate patients to notify clinic upon signs of infection

BRAT, bananas, rice, applesauce, toast; BTK, Bruton’s tyrosine kinase; CT, computed tomography; G-CSF, granulocyte colony stimulating factor.

## Data Availability

Not applicable.
